# Summer high temperature extremes over Northeastern China predicted by spring soil moisture

**DOI:** 10.1038/s41598-019-49053-9

**Published:** 2019-08-29

**Authors:** Jingyong Zhang, Zhanmei Yang, Lingyun Wu, Kai Yang

**Affiliations:** 1 Center for Monsoon System Research, Institute of Atmospheric Physics, Chinese Academy of Sciences, Beijing, 100029 China; 2 College of Earth and Planetary Sciences, University of Chinese Academy of Sciences, Beijing, 100049 China; 3 State Key Laboratory of Numerical Modeling for Atmospheric Sciences and Geophysical Fluid Dynamics (LASG), Institute of Atmospheric Physics, Chinese Academy of Sciences, Beijing, 100029 China

**Keywords:** Climate sciences, Atmospheric science

## Abstract

Current seasonal climate predictions mainly reside in the ocean anomalies. However, the prediction skills are generally limited over many extra-tropical land areas where the oceanic effects are relatively weak. In this study, we address the potential of preceding spring soil moisture condition to predict summer hot days over Northeastern China, a typical Northern Hemisphere mid-latitude region. The results show that spring soil moisture condition over Central-Eastern China is closely related with following summer hot days over Northeastern China for the period of 1979–2017. The statistical model based on the preceding spring soil moisture condition yields temporal cross-validated correlation skill of 0.57 for summer hot days over Northeastern China. The spatial pattern correlation skills of independent hindcast experiments for 2009–2017 are also high, ranging from 0.87 to 0.94. Our results can be easily applied to practical prediction of summer hot days over Northeastern China, and help to provide better climate services and reduce the detrimental effects of extreme heat over this extra-tropical region.

## Introduction

Accurate seasonal climate forecasts save lives, support agriculture and water resource managements, and avoid or reduce economic losses. It is commonly acknowledged that the ocean anomalies serve as the primary source of seasonal climate forecasts^1–3^. The tropical temperature and precipitation can be successfully predicted months in advance to a large extend, thanks to continuingly improved understanding of ocean-atmosphere interactions, particularly the El Niño-Southern Oscillation (ENSO) events^4–7^. However, over the many extra-tropical land areas, the ocean has relatively weak impacts, and the seasonal prediction skills are generally low in both mean climate and climate extremes^8–11^.

The slowly varying soil moisture takes a crucial role in modulating surface climate during the summer over some middle and high latitude land areas such as Eastern China via altering local surface heat fluxes and also changing regional atmospheric circulation^12–15^. In particular, dry soil moisture anomalies are recently highlighted to dominate the occurrence of summer extreme high temperature events associated with daily maximum surface temperature^16–19^, which exert many negative effects on human health, the economy, and the ecosystems^20–27^. Moreover, there are clear evidences that the preceding soil moisture or precipitation conditions provide potentially key sources for predictability of summer hot extremes^28–31^.

**Figure 1:**
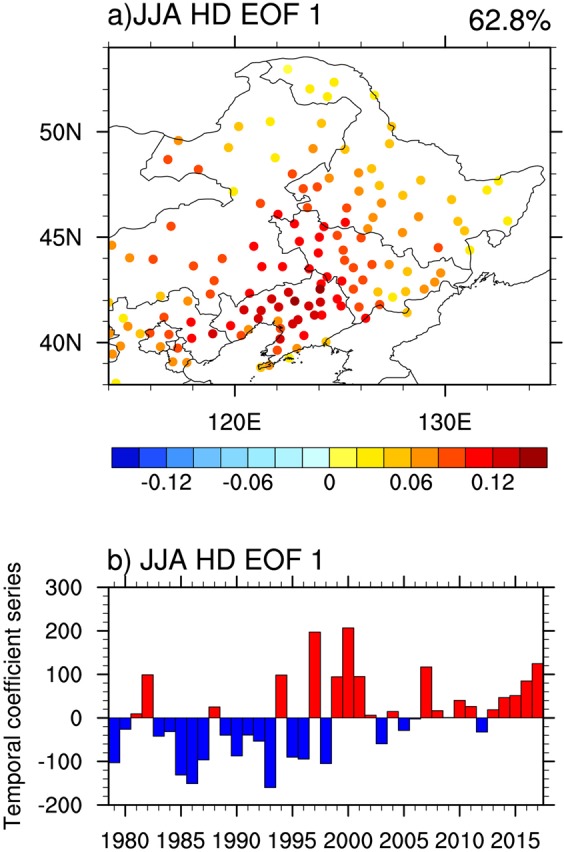
(**a**) Spatial pattern of the EOF first mode of summer (June-July-August) hot days during 1979–2017 at 136 stations over Northeastern China. The value above the figure indicates the fraction of variance explained by the first mode. The figure was created using NCAR Command Language (NCL) version 6.3.0 (10.5065/D6WD3XH5). (**b**) The temporal coefficient series of the EOF first mode from 1979 to 2017. The red and blue color indicate the positive and negative values.

Northeastern China, a typical Northern Hemisphere middle-latitude region, faces increasing impacts and risks of extreme heat events during the summer^32–39^. In this study, we investigate the role of spring (March-April-May) soil moisture condition in following summer (June-July-August) hot days over Northeastern China (Fig. 1a), and further explore the potential to use the preceding spring soil moisture information to predict summer hot days over this region.

We firstly used Empirical Orthogonal Function (EOF) analysis to check the spatial homogeneity of the summer hot days over Northeastern China. Next, we applied correlation analysis to investigate the relationship of spring soil moisture condition with summer hot days over Northeastern China, and identified the key region of spring soil moisture. Then, regression analysis was adopted to explore the possible underlying physical causes for the association of spring soil moisture condition over the key region with summer hot days over Northeastern China. Finally, we developed linear regression models to predict summer hot days over Northeastern China with preceding spring soil moisture condition over the key region. Prediction skills were tested by using leave-one-out cross-validation and independent hindcast experiments.

## Results

### Relationship of spring soil moisture condition with summer hot days over Northeastern China

**Figure 2:**
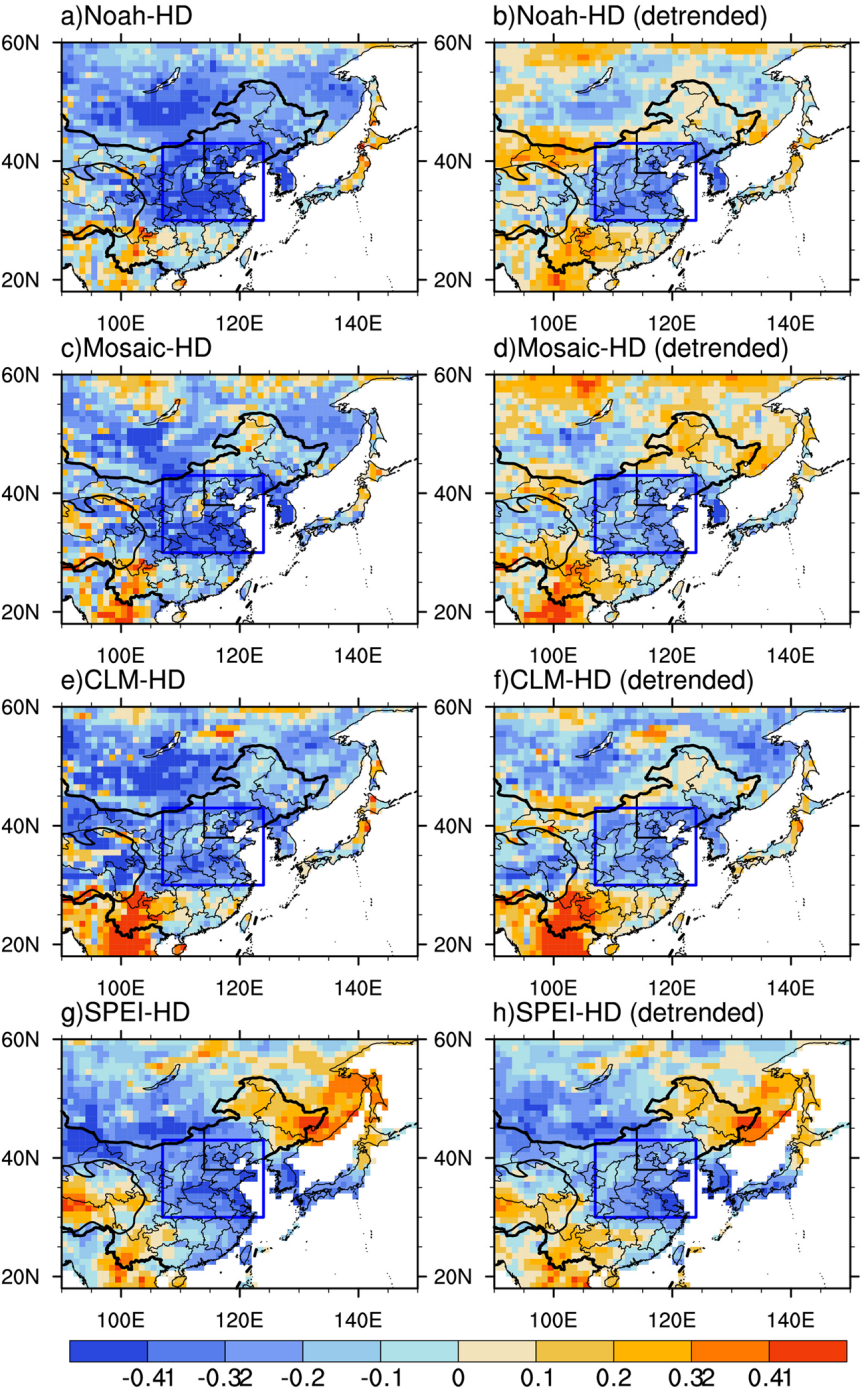
Spatial patterns of correlation coefficients between summer (June-July-August) hot days averaged over Northeastern China and spring (March-April-May) soil moisture conditions in (**a**,**b**) GLDAS-Noah (**c**,**d**) GLDAS-Mosaic (**e**,**f**) GLDAS-CLM and (**g**,**h**) SPEI as a proxy of soil moisture during 1979–2017 for original (left panel) and detrended (right panel) data. The strong correlation region, which is located in Central-Eastern China [107°E–124°E, 30°N–43°N], has been enclosed by the blue box. Straight black lines depict the boundaries of Northeastern China. Values of ±0.32 and ±0.41 represent that the correlations are significant at P < 0.05 and P < 0.01, respectively. The figure was created using NCAR Command Language (NCL) version 6.3.0 (10.5065/D6WD3XH5).

**Figure 3:**
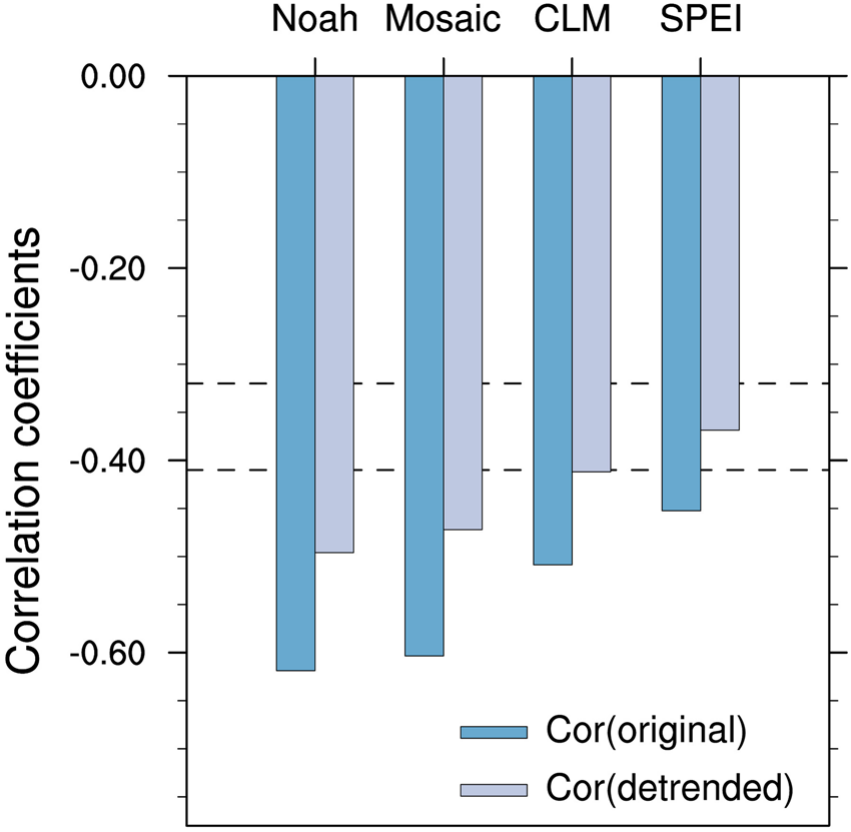
Correlation coefficients between summer (June-July-August) hot days averaged over Northeastern China and spring (March-April-May) soil moisture condition averaged over Central-Eastern China [107°E–124°E, 30°N–43°N] in GLDAS-Noah, GLDAS-Mosaic, GLDAS-CLM and SPEI as a proxy of soil moisture during 1979–2017. Blue and grey bars represent original and detrended time series, respectively. Below the two dash lines of –0.32 and –0.41, the correlations are significant at P < 0.05 and P < 0.01.

The EOF first mode of summer hot days at 136 stations over Northeastern China for 1979–2017, which can explain 62.8% of the total variance, indicates that spatial pattern of summer hot days is homogeneous (Fig. 1a). Furthermore, the time series of the EOF first mode are highly consistent with those of summer hot days averaged over Northeastern China (Fig. 1b). Figure 2 presents that summer hot days averaged over Northeastern China are largely negatively correlated with preceding spring soil moisture conditions from GLDAS-Noah, GLDAS-Mosaic, and GLDAS-CLM, and SPEI as a proxy of soil moisture over Eastern Asia for original and detrended data of 1979–2017. The consistent strong correlations appear over the region of Central-Eastern China enclosed by the blue box [107°E–124°E, 30°N–43°N] with most values significant at the 0.05 level for both original and detrended different datasets. We further calculate correlation coefficients of the spring soil moisture conditions averaged over Central-Eastern China with summer hot days averaged over Northeastern China for 1979–2017 (Fig. 3). The correlation coefficients of different GLDAS soil moisture datasets with summer hot days are all significant at the 0.01 level for both original and detrended data. For SPEI, the correlation coefficients are significant at the 0.01 and 0.05 levels, respectively. We also repeat our analyses using the data for 1979–2008, and find that the conclusions agree well with those for 1979–2017 (Figs S1 and S2). The agreements among original and detrended different soil moisture condition datasets for both 1979–2017 and 1979–2008 indicate the robustness of the close association of spring soil moisture condition over Central-Eastern China with summer hot days over Northeastern China.

**Figure 4:**
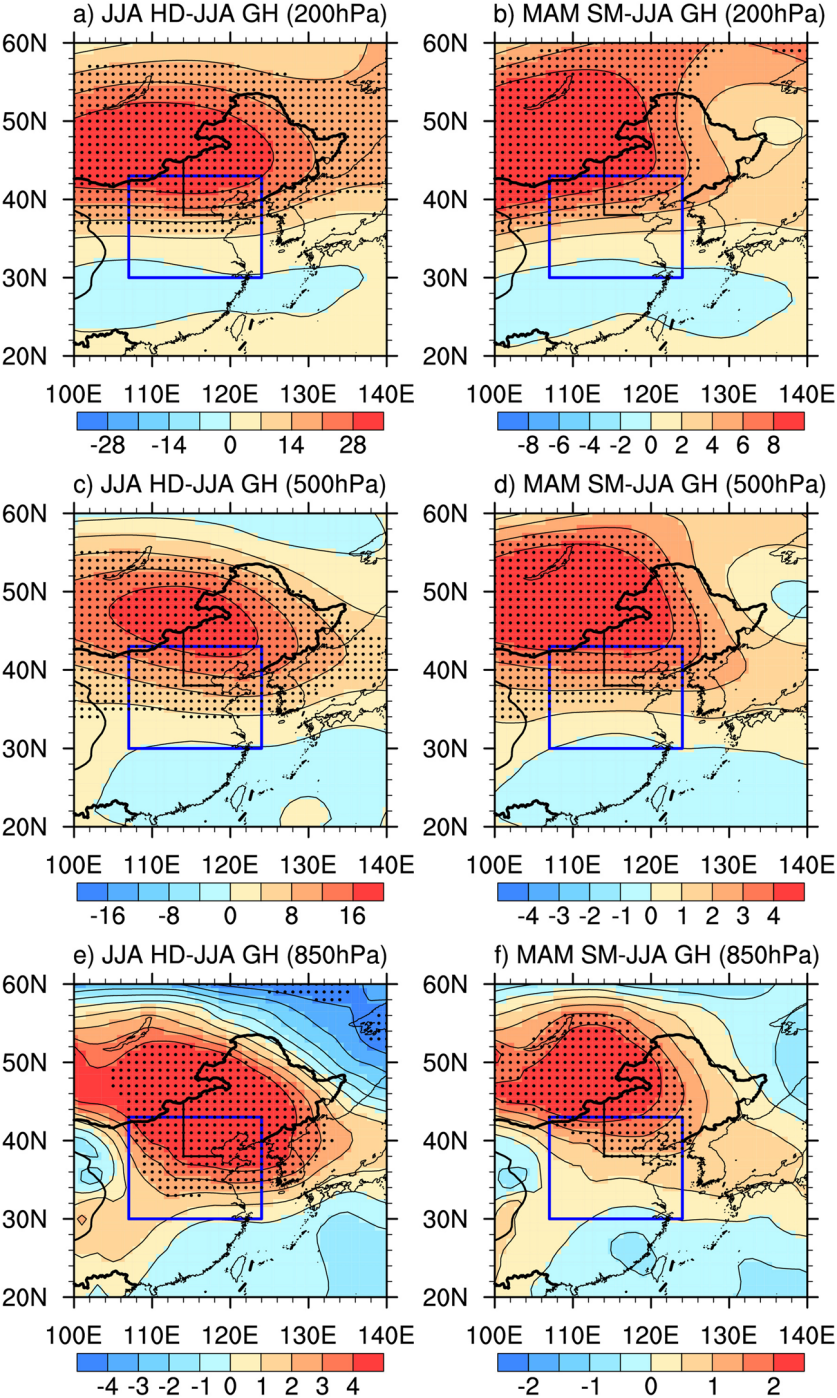
Linear regression coefficients of summer (June-July-August) geopotential heights in (**a**,**b**) 200 hPa, (**c**,**d**) 500 hPa, (**e**,**f**) 850 hPa against summer (June-July-August) hot days (in gpm/day, left panel) and spring (March-April-May) soil moisture condition(multiplies -1) averaged over Central-Eastern China [107°E–124°E, 30°N–43°N] in GLDAS-Noah (in gpm/mm, right panel) during 1979–2017. Stippling denotes areas where the regression coefficients are significant at P < 0.05. The blue box denotes the key region [107°E–124°E, 30°N–43°N], where is located in Central-Eastern China. Straight black lines depict the boundaries of Northeastern China used in this study. The figure was created using NCAR Command Language (NCL) version 6.3.0 (10.5065/D6WD3XH5).

**Figure 5:**
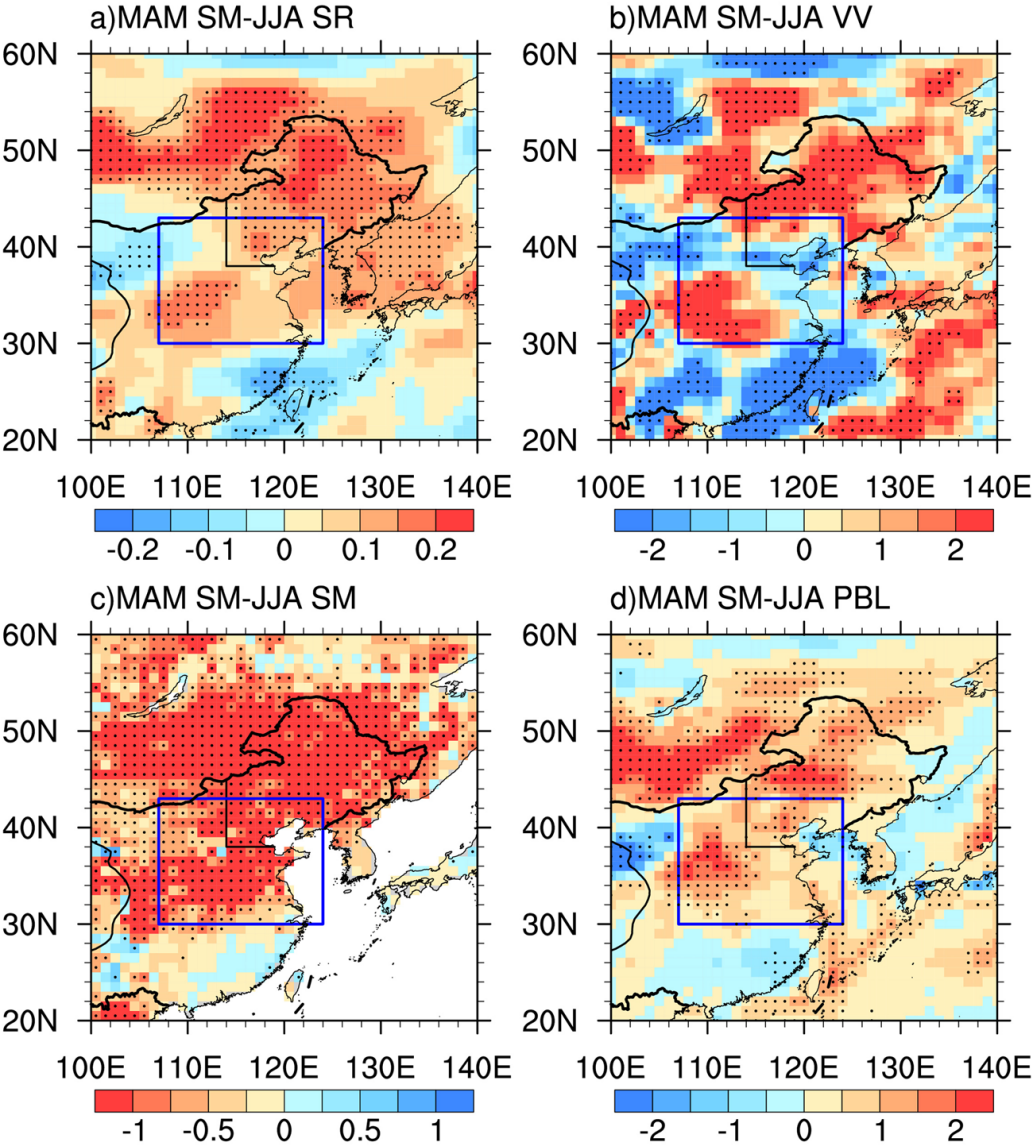
Linear regression coefficients of summer (June-July-August) (**a**) surface downward solar radiation (in W/m^2^) (**b**) 500 hPa vertical velocity (in 10^−4^ Pa/s) (**c**) soil moisture (in mm) and (**d**) planetary boundary layer height (in m) against spring (March-April-May) soil moisture condition (in mm, multiplies -1) averaged over Central-Eastern China [107°E–124°E, 30°N–43°N] in GLDAS-Noah during 1979–2017. Stippling denotes areas where the regression coefficients are significant at P < 0.05. The blue box denotes Central–Eastern China [107°E–124°E, 30°N–43°N]. Straight black lines depict the boundaries of Northeastern China. The figure was created using NCAR Command Language (NCL) version 6.3.0 (10.5065/D6WD3XH5).

Regression analysis shows that summer hot days correspond well with higher summer geopotential heights over Northeastern China (Fig. 4a,c,e), which is supported by previous studies^33,40,41^. According to dry spring soil moisture anomalies over upstream Central-Eastern China, there are significant and positive summer 200 hPa, 500 hPa and 850 hPa geopotential height anomalies over Mongolia and Northeastern China (Fig. 4b,d,f). There are evidences that spring anomalies of land surface conditions can stimulate summer downstream changes of atmospheric circulations including geopotential heights^42–47^. For example, Koster *et al*. demonstrated that preceding soil moisture dryness can trigger positive geopotential height anomalies over nearby non-local region based on numerical experiments^44^. Similar mechanisms may provide possible physical explanation for the link of spring soil moisture anomalies over Central-Eastern China to following summer geopotential height anomalies over downstream Northeastern China. The positive summer geopotential height anomalies associated with dry spring soil moisture anomalies tend to lead to more downward solar radiation (Fig. 5a) and stronger sinking motion (Fig. 5b), which may subsequently enhance surface heat fluxes and subsidence warming in summer over Northeastern China, respectively. In addition, decreased precipitation associated with increased geopotential heights may result in significant soil dryness over Northeastern China (Fig. 5c), increasing local sensible heat and the entrainment of heat into planetary boundary layer (Fig. 5d). These changed processes possibly induced by dry spring soil moisture condition over upstream Central-Eastern China together may cause more summer high temperature extremes over Northeastern China. Due to complex dynamical and thermodynamic processes involved, numerical experiments need to be further performed to better understand the underlying mechanisms explaining the close association of spring soil moisture condition over Central-Eastern China with summer hot extremes over Northeastern China.

### Prediction of summer hot days over Northeastern China

**Figure 6:**
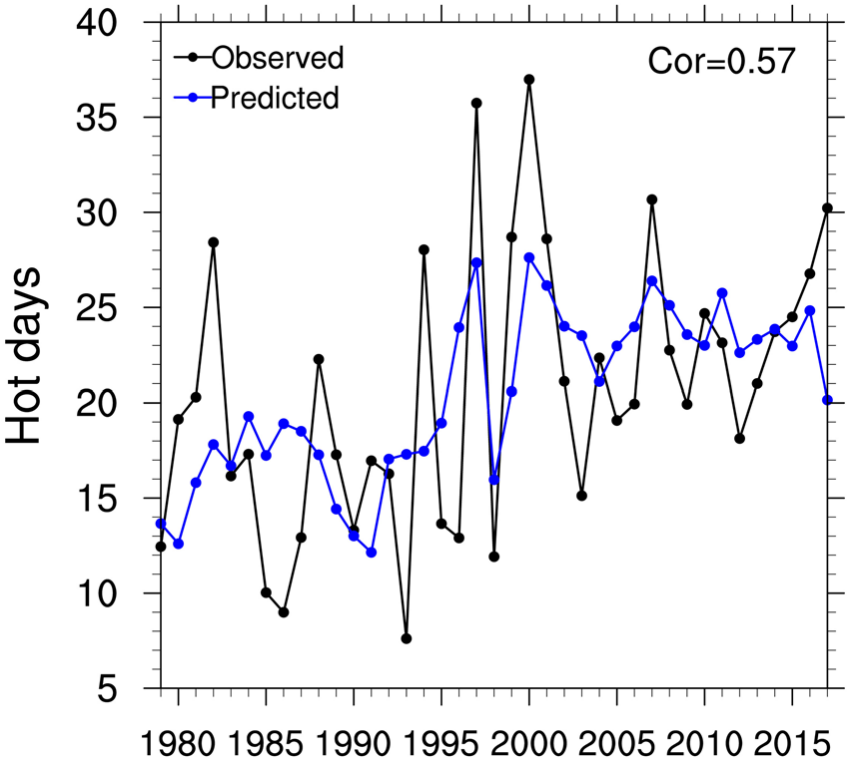
Time series of summer (June-July-August) hot days (in days) averaged over Northeastern China from 1979 to 2017. The black and blue lines represent the observation and prediction according to leave-one-out cross-validation, respectively.

As described above, the summer hot days over Northeastern China have strong correlations with preceding spring soil moisture condition over Central-Eastern China. Here, we use soil moisture from GLDAS-Noah which has the highest correlation with summer hot days among three GLDAS soil moisture data and SPEI, as the predictor, and further establish linear regression models for summer hot days averaged over Northeastern China. Figure 6 shows that the correlation coefficient between the observed summer hot days and leave-one-out cross-validation estimates over Northeastern China is 0.57 for 1979–2017. And the result indicates that about one third (32.5%) of the total variance of summer hot days over Northeastern China can be predicted by the statistical models based on the spring soil moisture condition.

**Figure 7:**
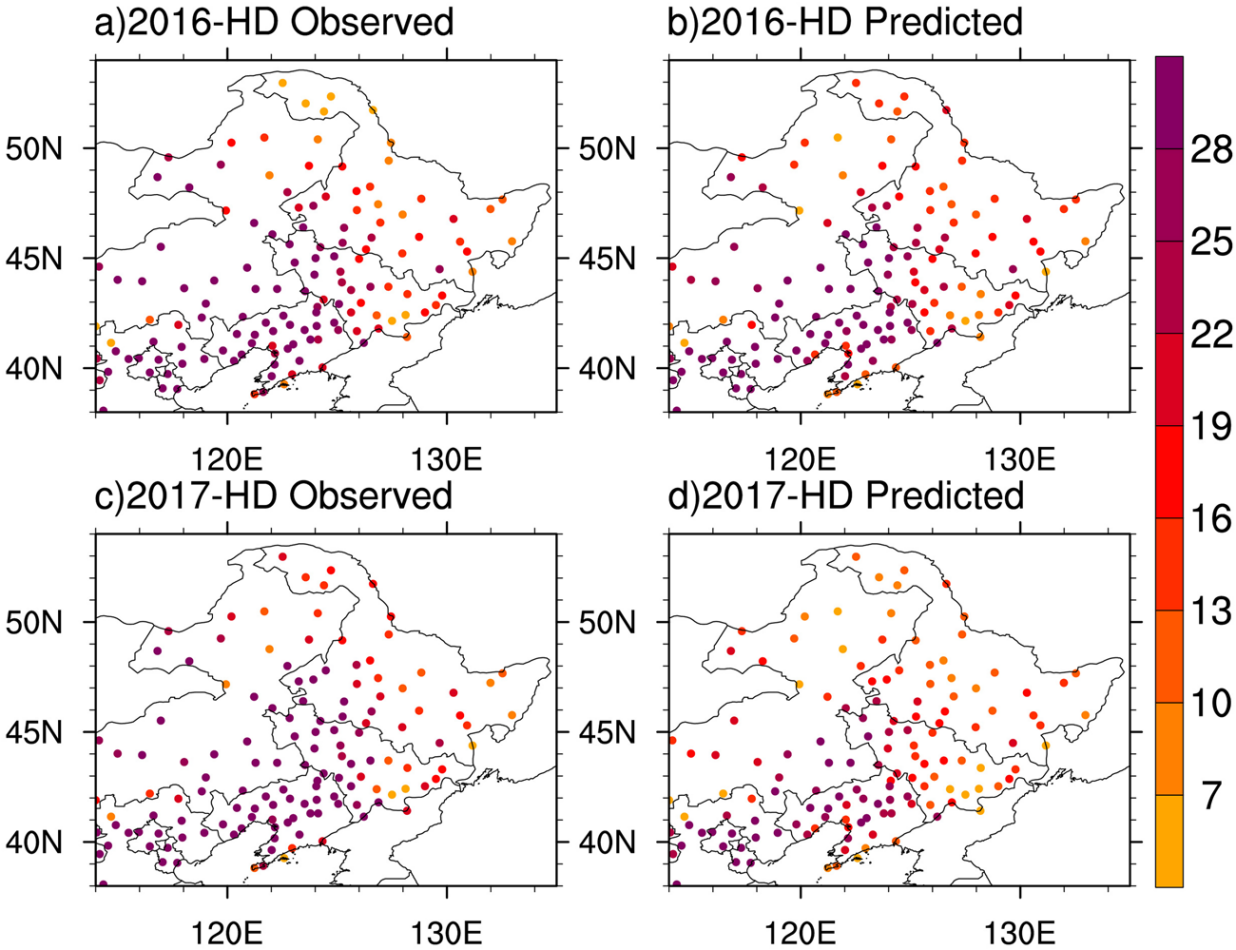
Maps of summer (June-July-August) hot days (in days) over Northeastern China for (**a**,**b**) 2016 and (**c**,**d**) 2017: observations (left panel) and predictions (right panel). The figure was created using NCAR Command Language (NCL) version 6.3.0 (10.5065/D6WD3XH5).

**Figure 8:**
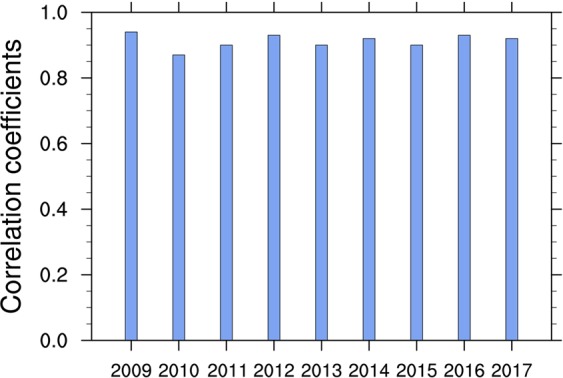
Spatial pattern correlation coefficients between observed and predicted summer (June-July-August) hot days over Northeastern China from 2009 to 2017.

Then, we predict spatial patterns of summer hot days over Northeastern China with independent hindcast experiments for 2009 to 2017 by using 30-year-moving prediction models for each of 136 stations. Figure 7 presents examples of 2016 and 2017 for the observed and predicted summer hot days at 136 stations over Northeastern China. The observed and predicted summer hot days generally show the consistent spatial pattern with a obvious south-to-north gradient. We also calculate the spatial pattern correlation coefficients between the observed and predicted summer hot days over Northeastern China for 2009–2017 (Fig. 8). The correlation coefficients range from 0.87 to 0.94, indicating that the statistic models we developed have high prediction skills for spatial patterns of summer hot days over Northeastern China.

## Conclusions and Discussion

Northeastern China which is located in middle latitudes of Northern Hemisphere, is increasingly influenced by extreme high temperature events during the summer. However, current limited skills of seasonal climate prediction impede our ability to deal with these heat events. In this study, we identify that preceding spring soil moisture conditions over Central-Eastern China have robust and negative associations with summertime hot days over Northeastern China for 1979–2017. These close relationships point to that spring soil moisture condition can be used as the potentially important predictor for summer hot days over Northeastern China.

Dry soil moisture condition in spring over upstream Central-Eastern China corresponds well to summer geopotential heights over Northeastern China. The increased geopotential height may subsequently rise downward solar radiation reaching land surface and enhance subsidence warming, leading to warmer surface air temperature and more hot days over Northeastern China during the summer. In addition, the increased geopotential height tends to result in less precipitation and drier soil over Northeastern China, which may subsequently cause stronger local surface sensible heat fluxes and enhance the entrainment of warm air into the planetary boundary layer. These possible physical processes directly and indirectly resulted from dry soil moisture condition favor the occurrence of summer hot days over Northeastern China.

Spring soil moisture condition over Central-Eastern China is further used to predict hot days over Northeastern China during the following summer. The statistical models developed with spring soil moisture over Central-Eastern China yield significant leave-one-out cross-validated correlation skill of 0.57 for the prediction of summer hot days averaged over Northeastern China for the period of 1979–2017. Independent hindcast experiments for 2009–2017 show that the pattern correlation skills range from 0.87–0.94. These results indicate that summer hot days over Northeastern China can be largely predicted with preceding spring soil moisture over Central-Eastern China.

This study highlights the importance of spring soil moisture condition over upstream Central-Eastern China for predicting summer high temperature extremes over Northeastern China. Meanwhile, some limitations exist. These proposed physical mechanisms underlying the close association of spring soil moisture condition with following summer high temperature extremes over Northeastern China need to be further tested by model simulations in the future. In addition to soil moisture condition, sea surface temperature, soil temperature, vegetation, sea ice among others have the potential for summer high temperature extreme prediction^47,48^. For example, Zhang *et al*. made the relative accurate prediction of summer high temperature extremes over the source region of the ancient Silk Road in China by taking spring soil temperature, sea surface temperature and large-scale climate indices as preceding precusors^49^. In particular, the ENSO is well known to largely control the interannual climate variability on the global scale. However, our preliminary analysis does not identify significant correlations of summer high temperature extremes over Northeastern China with preceding ENSO indices, which are subject to additional investigation. Current dynamical models generally show very low skills in forecasting high temperature extremes, and realistic soil moisture initialization and improved representation of soil processes have the potential to enhance the monthly-to-seasonal forecast skills in dynamical models^17,50^. Our findings are expected to facilitate the improvement of practical seasonal forecasting for summer hot days over Northeastern China.

## Methods

### Data preparation

The daily surface air temperature records for 699 stations in China were obtained from the China Meteorological Data Service Center. In this study, we chose 136 stations over Northeastern China with complete records of daily maximum surface air temperature in summer (June-July-August) for 1979–2017 (Fig. 1a). Soil moisture data were obtained from the Global Land Data Assimilation System (GLDAS) version 1.0 at a resolution of 1.0° × 1.0°, which were produced by driving offline land surface models with integrated observation based data^51^. Spring (March-April-May) soil moisture data from GLDAS-Noah, GLDAS-Mosaic, and GLDAS-CLM for 1979–2017 were applied with soil layers of 0.1–1 m, 0.02–1.5 m, 0.09–1.38 m, respectively. We took the standardized precipitation evapotranspiration index (SPEI) data at a 3-month time scale as a proxy of soil moisture^52^ to test if our results depend on the choice of soil moisture datasets.

The monthly geopotential heights at 200 hPa, 500 hPa and 850 hPa, surface downward solar radiation, 500 hPa vertical velocity and planetary boundary layer height were taken from the European Center for Medium-range Weather Forecasts (ECMWF)’s ERA-interim reanalysis dataset^53^.

### Hot day definition and EOF analysis

A day in summer (June-July-August) was defined as a hot day in which daily maximum temperature was equal to or above 30 °C. EOF analysis, which can extract the principal spatial and temporal characteristics from the variable, is applied to check the spatial homogeneity of the summer hot days at 136 stations over Northeastern China.

### Correlation with spring soil moisture

The correlation analysis was employed to examine the relationship between summer hot days averaged over Northeastern China and spring soil moisture over East Asia for 1979–2017. We also conducted the correlation analysis after removing the linear trends of data. Student’s *t*-test was used to identify the key region of statistical significance for both original and detrended data. As shown in Fig. 2, the identified key region of spring soil moisture is located in Central-Eastern China. In addition, we also tested the robustness of the key region by the correlation analysis of the data for 1979–2008 (Figs S1 and S2).

### Physical process analysis

Regression analysis was applied to explored the possible physical processes associated with the link between summer hot days over Northeastern China and spring soil moisture conditions over Central-Eastern China. We calculated the regression coefficients of 200 hPa, 500 hPa and 850 hPa geopotential heights, surface downward solar radiation, 500 hPa vertical velocity, soil moisture, and planetary boundary layer height in summer against spring soil moisture averaged over Central-Eastern China for 1979–2017.

### Prediction model development and evaluation

We developed linear regression prediction model for summer hot days over Northeastern China based on the identified spring soil moisture over Central-Eastern China. We used leave-one-out cross-validation to evaluate how well the statistical models perform. Each time, one year observation from 1979–2017 was chosen as the validation set, and the remaining observations were used as the training set. Both spring soil moisture and summer hot days for each prediction year are dropped out when statistical model is developed. This cross-validation process was repeated 39 times until all predictions were conducted. We used the correlation coefficient between the observed and the corresponding cross-validation estimates to measure the prediction skill.

### Spatial pattern prediction

Finally, we conducted independent hindcast experiments of summer hot days at 136 stations over Northeastern China from 2009 to 2017. The 30-year-moving prediction models at each station were established based on the identified spring soil moisture over Central-Eastern China. For example, we used the data for 1979–2008, 1980–2009 and 1981–2010 to establish the prediction models for summer hot days at each station in 2009, 2010 and 2011, respectively. The observed and predicted summer hot days at 136 stations in 2016 and 2017 were shown in Fig. 7. The correlation coefficients between the observed and predicted spatial patterns of summer hot days for 2009–2017 were computed to evaluate the prediction skills (Fig. 8).

## Supplementary information

Supplementary Information

## Data Availability

The data sets within the article are available from authors upon reasonable request.
