# Supplementary material for: Summer high temperature extremes over Northeastern China predicted by spring soil moisture
Source: Sci Rep. 2019 Aug 29;9:12577. doi: 10.1038/s41598-019-49053-9 (PMC6715730; doi:10.1038/s41598-019-49053-9)
Supplement: Supplementary file 1 — Supplementary Information [file 41598_2019_49053_MOESM1_ESM.doc]

**Supplementary Information for**

**Summer high temperature extremes over Northeastern China predicted by spring soil moisture**

Jingyong Zhang^1,2^, Zhanmei Yang^1,2^, Lingyun Wu^3^ & Kai Yang^3^

^1^Center for Monsoon System Research, Institute of Atmospheric Physics, Chinese Academy of Sciences, Beijing 100029, China

^2^College of Earth and Planetary Sciences, University of Chinese Academy of Sciences, Beijing 100049, China

^3^State Key Laboratory of Numerical Modeling for Atmospheric Sciences and Geophysical Fluid Dynamics (LASG), Institute of Atmospheric Physics, Chinese Academy of Sciences, Beijing 100029, China

Correspondence

Prof. Jingyong Zhang

Center for Monsoon System Research, Institute of Atmospheric Physics, Chinese Academy of Sciences, Beijing 100029, China

Email: [zjy@mail.iap.ac.cn](mailto:zjy@mail.iap.ac.cn)

**This file includes**

Figure. S1

Figure. S2


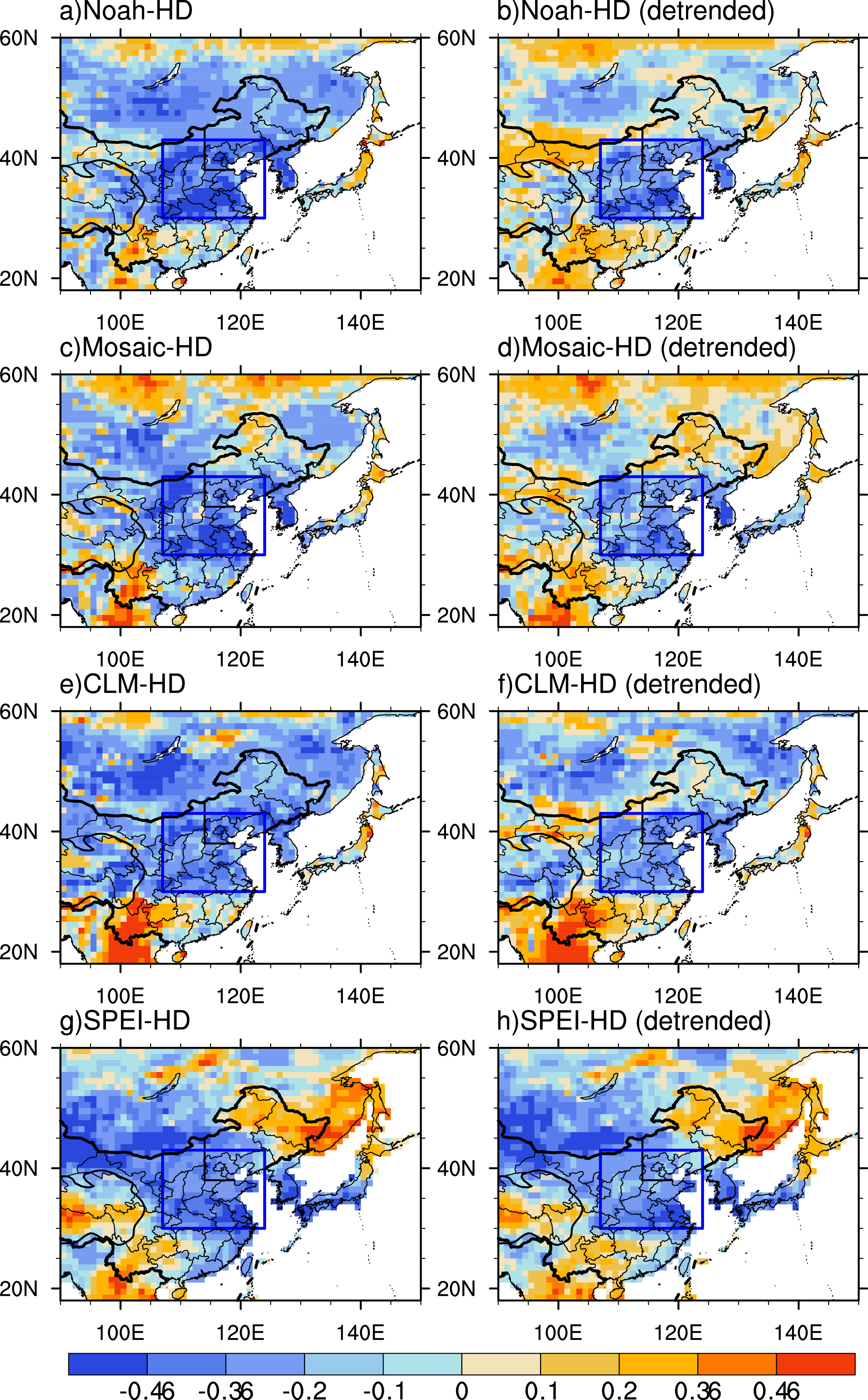


**Figure S1**. Spatial patterns of correlation coefficients between summer (June-July-August) hot days averaged over Northeastern China and spring (March-April-May) soil moisture condition in (a, b) GLDAS-Noah (c, d) GLDAS-Mosaic (e, f) GLDAS-CLM and (g, h) SPEI as a proxy of soil moisture during 1979–2008 for original (left panel) and detrended (right panel) data. The strong correlation region, which is located in Central-Eastern China [107°E–124°E, 30°N–43°N], has been enclosed by the blue box. Straight black lines depict the boundaries of Northeastern China. Values of ±0.36 and ±0.46 represent that the correlations are significant at P<0.05 and P<0.01, respectively. The figure was created using NCAR Command Language (NCL) version 6.3.0 (http://dx.doi.org/10.5065/D6WD3XH5).


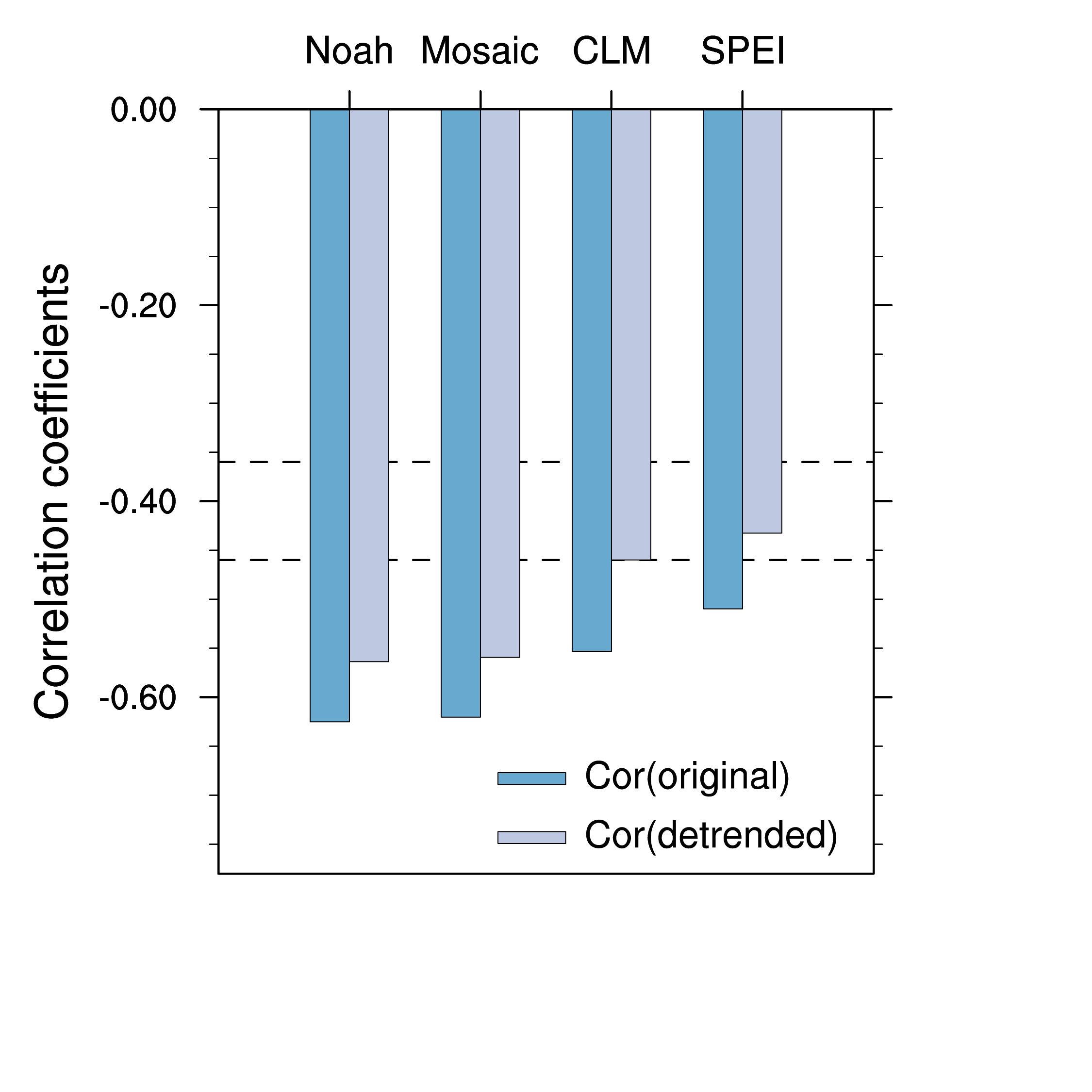


**Figure S2**. Correlation coefficients between summer (June‑July-August) hot days averaged over Northeastern China and spring (March-April-May) soil moisture condition averaged over Central-Eastern China [107°E–124°E, 30°N–43°N] in GLDAS-Noah, GLDAS-Mosaic, GLDAS-CLM and SPEI as a proxy of soil moisture during 1979–2008. Blue and grey bars represent original and detrended time series, respectively. Below the two dash lines of -0.36 and -0.46, the correlations are significant at P<0.05 and P<0.01.
